# Comparing the posterolateral and the direct lateral approach for cemented hemiarthroplasty after femoral neck fracture: a cost-effectiveness analysis

**DOI:** 10.2340/17453674.2025.45056

**Published:** 2025-12-18

**Authors:** Jonas L ESSER, Maria C J M TOL, Nienke W WILLIGENBURG, Ariena J RASKER, Taco GOSENS, Martijn G M SCHOTANUS, Hanna C WILLEMS, Martin J HEETVELD, J Carel GOSLINGS, Johanna M VAN DONGEN, Rudolf W POOLMAN

**Affiliations:** 1 Department of Health Sciences, Vrije Universiteit, Amsterdam; 2 Department of Orthopedic Surgery, Joint Research, OLVG Hospital, Amsterdam; 3 Department of Orthopedics and Trauma Surgery, ETZ, Tilburg; 4 Department of Medical and Clinical Psychology, Tilburg University, Tilburg; 5 Department of Orthopedic Surgery & Traumatology, Zuyderland Medical Center, Heerlen, Sittard-Geleen; 6 School of Care and Public Health Research Institute, Faculty of Health, Medicine and Life Science, Maastricht University; 7 Department of Internal Medicine and Geriatrics, Amsterdam UMC, Amsterdam; 8 Department of Trauma Surgery, Spaarne Gasthuis, Haarlem; 9 Department of Trauma Surgery, OLVG Hospital, Amsterdam; 10 Department of Orthopedic Surgery, LUMC, Leiden, the Netherlands

## Abstract

**Background and purpose:**

The 2 most common surgical approaches in hemiarthroplasty for hip fracture treatment are the posterolateral and the direct lateral approach. We aimed to estimate the relative cost-effectiveness of these approaches.

**Methods:**

We conducted an economic evaluation alongside a randomized controlled superiority trial for 6 months. The trial included 555 patients over 18 years of age with an acute femoral neck fracture. The effectiveness outcome used was quality-adjusted life years (QALYs), assessed using the EQ-5D-5L. Costs were measured through self-reported questionnaires administered at baseline, after 3 months, and after 6 months. We dealt with missing data through multiple imputation and analyzed the imputed datasets by comparing group means in costs and QALYs. A secondary analysis included adjustment for baseline imbalances through linear regression.

**Results:**

The estimated average treatment effect on the QALYs was 0.02 (95% confidence interval [CI] –0.006 to 0.046). From the healthcare and societal perspective, we found a non-significant average treatment effect on costs of 1,508 (CI –1,744 to 4,760) and 1,583 (CI –1,972 to 5,137), respectively. The probability of cost-effectiveness was 10% at a willingness-to-pay of zero, and then slowly increased to around 50% for higher willingness-to-pay values.

**Conclusion:**

We found no conclusive evidence of any differences between the surgical approaches with respect to costs, QALYs, and cost-effectiveness. We therefore suggest that, from an economic viewpoint, the 2 surgical approaches should be treated as interchangeable.

The number of hip fractures is expected to rise substantially in the coming decades [1]. Given their significant impact on health-related quality of life (HRQoL) and healthcare costs [2-4], effective and cost-effective treatment is essential. Hemiarthroplasty is a commonly used treatment for femoral neck fractures. The most common approaches are the direct lateral approach (DLA) and the posterolateral approach (PLA) [5].

Until recently, the evidence regarding the comparative effectiveness of DLA versus PLA was derived mainly from observational studies [6,7]. A 2021 systematic review suggested that PLA may provide advantages over DLA with respect to HRQoL, abductor insufficiency, and gait-related impairments [8]. However, these potential benefits of PLA may be outweighed by a higher risk of dislocation and reoperation compared with both DLA and the direct anterior approach [9]. More recently, a large randomized controlled trial found no differences in HRQoL, pain, activities of daily living (ADL) independence, or mobility between the approaches, but did report a significantly higher rate of dislocation and reoperation after PLA [10].

Reoperations are significant drivers of costs [11], which may make PLA less cost-effective than DLA. However, direct evidence on the 2 treatments’ relative cost-effectiveness is currently lacking. Cost-effectiveness analyses show whether health gains are achieved in proportion to the resources used, thereby informing policy, reimbursement, and prioritization within constrained healthcare budgets [12]. Even when initial treatment costs are similar, as in the case of PLA and DLA, total healthcare and societal costs may diverge considerably if recovery is quicker or more complete, reducing downstream productivity losses and healthcare use. We aimed to assess the relative cost-effectiveness of PLA and DLA [13], measured as quality-adjusted life years (QALYs), while costs are considered from both a healthcare and a societal perspective. 

## Methods

### Study design and procedures

This economic evaluation is based on a multicenter randomized controlled trial (RCT) with a superiority design [14]. We recruited patients in 5 Dutch hospitals, where the local surgeons could perform both PLA and DLA. We screened all patients admitted to the recruited hospitals for eligibility and invited them to participate in the RCT before the surgery. Inclusion criteria were: ≥ 18 years, acute femoral neck fracture (≤ 7 days), cemented hemiarthroplasty as recommended treatment, and written informed consent. Multi-trauma patients (Injury Severity Score > 15), and patients with secondary surgery of the hip or pathological fractures were excluded. After informed consent, we randomly assigned each patient to either the PLA or DLA group using CASTOR EDC (www.castoredc.com), with equal probabilities. Patients, surgeons, and other medical personnel were not blinded. The study is reported according to the recommendations in Sanders et al. [13].

### Interventions

#### Posterolateral approach (PLA)

The external rotators and piriformis are dissected in the PLA group, and a posterior capsulotomy is performed. The gluteus medius and vastus lateralis muscles are preserved. The surgeon’s preference determined whether the piriformis was spared or reattached.

#### Direct lateral approach (DLA)

In the DLA group, the anterior insertion of the gluteus medius is released proximally, and the fibers of the vastus lateralis are divided. An anterior capsulotomy is performed while preserving the posterior capsule.

### Measurements

The effectiveness outcome was quality-adjusted life years (QALYs), measured with the EQ-5D-5L at baseline (that is, before the surgery), as well as 3 months and 6 months after the surgery [15]. It should be noted that at the time of recruitment all patients had already sustained a femoral neck fracture; “baseline” thus does not represent the pre-fracture health state. The patients’ EQ-5D-5L health states were converted into utility scores ranging from –0.446 (worse than dead) to 1 (optimal health), with a score of 0 indicating death, based on the Dutch utility tariff [16]. QALYs were calculated as a weighted average of the reported utility scores (see Appendix 1).

Resource-use information was obtained by questionnaires administered 1, 3, and 6 months after the surgery. The questionnaires covered the following categories:

- initial surgery;
- follow-up surgeries due to complications;
- primary healthcare use (e.g., general practitioner);
- secondary healthcare use (e.g., specialists, hospital expenses);
- medication use (over-the-counter and prescription-only drugs);
- unpaid productivity losses (i.e., volunteer work);
- informal care (i.e., care by family members).

In addition to the above cost categories, it is customary to include work-related costs (e.g., absenteeism). However, this was not applicable in the present study, as all patients were retired and therefore not employed at the time of injury.

All resource use was valued in accordance with the Dutch Manual of Costing [17], with all costs being expressed in euros (2021). Total costs were estimated from the healthcare perspective (only including surgery costs, primary healthcare costs, secondary healthcare costs, and medication costs), and the societal perspective (including all of the cost categories listed above).

### Sample size

To detect a minimally clinically important difference (MCID) of 0.08 in the EQ-5D-5L utility scores [18], from which QALYs were derived, we required a sample size of 555 patients. This was based on a 2-sided significance level (α) of 0.05 with 80% power, a standard deviation of 0.3, and a 20% loss to follow-up after 6 months [14].

### Statistics

Average treatment effects on costs and QALYs were estimated in 2 ways:

1. Crude analysis, comparing mean costs and QALYs between the 2 surgical approaches.
2. Adjusted analysis, where we used 2 separate linear regressions to estimate the effects on costs and QALYs, respectively. Here, we adjusted for the baseline cost and utility measurements.

Analyses were performed from the healthcare and societal perspective. We used bootstrapping to estimate the sampling variance of all statistics of interest.

Given that the data is entirely composed of questionnaires filled out by elderly patients (or their proxies), we expected to encounter large numbers of partially missing observations. Therefore, we used multivariate imputation by chained equations (MICE) to deal with missing data. Imputation works by generating artificial values, which then replace the missing values. We specifically used the predictive mean matching (PMM) method [19] as implemented in the mice software package [20]. We imputed a total of 100 datasets. The imputation model included the baseline variables (see Table 1), and variables that enter into the calculation of the outcome variables. For each imputed dataset, we performed the analyses described below, after which we pooled the point estimates and standard errors using Rubin’s rules [21]. Confidence intervals were computed based on a normal approximation, using the estimated variances, as this approach has been shown to perform well in a recent simulation study [22].

**Table 1:** Baseline characteristics. Values are count (%) unless otherwise specified

Item	PLA group (n = 272)	DLA group (n = 283)	Standardized difference
Age, mean (SD)	82 (8)	82 (7)	0
Female sex	172 (63)	172 (61)	0.03
BMI, mean (SD)	24.7 (4.2)	24.2 (4.1)	0.09
ASA I	4 (1.5)	8 (2.8)	–0.06
ASA II	86 (32)	107 (38)	–0.09
ASA III	171 (63)	158 (56)	0.10
ASA IV	11 (4.0)	10 (3.5)	0.02
Impaired mobility	166 (61)	175 (62)	–0.02
Dependent living status (e.g., nursing home)	52 (19)	64 (23)	–0.07
Quality of life, mean (SD) (EQ-5D utility score)	0.389 (0.358)	0.333 (0.366)	0.11

ASA = American Society of Anesthesiologists Physical Status classification, BMI = body mass index, SD = standard deviation,

### Cost-effectiveness analysis

For the cost-effectiveness analysis, we estimate the average treatment effects on costs and QALYs based on the observed data. These were combined into a utility function, Net monetary benefit (NMB), which informed the implementation decision: if the NMB was positive, PLA was considered cost-effective and should be implemented, and vice versa if the NMB was negative. Incremental cost-effectiveness ratios (ICERs) were calculated by dividing the estimated effect on costs by the estimated effect on the QALYs. We plotted bootstrapped cost-effect pairs on a cost-effectiveness plane to visually inspect the uncertainty surrounding the estimates [23]. A cost-effectiveness acceptability curve was provided to illustrate the probability of PLA being cost-effective at different levels of willingness-to-pay. In the Netherlands, decision-makers usually apply thresholds of €20,000, €50,000, and €80,000 per QALY, depending on the severity of the disease [17]. See also Appendix 1.

### Ethics, registration, data sharing plan, funding, and disclosures

The clinical trial was registered at ClinicalTrials.gov (identifier: NCT04438226) before the start of patient enrollment. The study received approval from both the local and Medical Ethics Committee (METC) under number NL63378.100.17 and was carried out in accordance with the principles of the Declaration of Helsinki, as revised in Seoul and Fortaleza (64th WMA General Assembly, October 2013) [24]. It also adhered to the Medical Research Involving Human Subjects Act (WMO) and all other relevant laws, regulations, and guidelines. In each participating hospital, the study protocol was submitted to the local research ethics board for review and approval.

All study data will be stored and maintained for 15 years at the initiating hospital (OLVG). We participate in data sharing in accordance with the FAIR (Findability, Accessibility, Interoperability, and Reuse) principles, considering European privacy regulations and guidelines, and the data is available upon reasonable request. Metadata and other information is available under https://doi.org/10.34894/K99WGS.

The trial was funded by the Dutch Organisation for Health Research and Development (ZonMw; grant numbers 843004112 and 10330112010006). None of the authors report any conflicts of interest. Complete disclosure of interest forms according to ICMJE are available on the article page, doi: 10.2340/17453674.2025.45056

## Results

**Figure 1:**
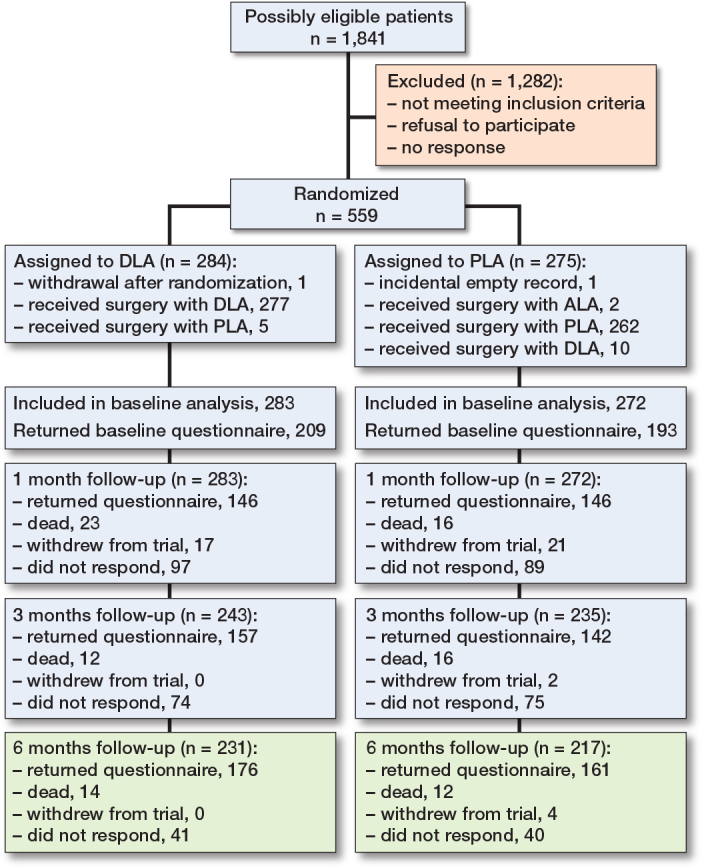
Patient flowchart, adapted from Tol et al. [10]. ALA = anterolateral approach.

Between February 2018 and January 2022, 555 patients were included (272 PLA and 283 DLA) (Figure 1). Regarding the patients’ baseline characteristics per treatment group the groups were in general well balanced, with no stark differences (Table 1).

### Missing data and imputation

72 out of 555 observations were complete, with respect to all variables of interest for this study. All patients had age and sex baseline measurements recorded. For all other baseline variables, missing values were present; the highest missingness proportion here was BMI, missing for 24% of patients. For answers to the EQ-5D-5L questionnaire, the missingness proportions at baseline, 3 months, and 6 months after the surgery were 27%, 46%, and 39%, respectively. For the cost questionnaires, the missingness proportions at 1 month, 3 months, and 6 months after the surgery were 40%, 32%, and 21%, respectively. Hence, all the following tables and figures are based on imputed data.

### Utility scores and cost variables

For both treatment groups, the EQ-5D-5L utility scores at 3 months and 6 months were much higher than at baseline, indicating that both treatments effectively improve the patients’ quality of life (Table 2). The utilities and QALYs were slightly higher in the PLA group (Table 2). Note, however, that the baseline utility was also higher in the PLA group, and the differences in Table 2 are not adjusted for that.

**Table 2:** Estimated group means with standard errors based on multiple imputations and differences in the disaggregated outcome variables with 95% confidence intervals (CI)

Variable	PLA group (n = 272)	DLA group (n = 283)	Difference (CI)
EQ–5D utility score			
3 months	0.530 (0.024)	0.482 (0.023)	0.047 (–0.019 to 0.078)
6 months	0.500 (0.024)	0.489 (0.024)	0.012 (–0.054 to 0.078)
QALY	0.244 (0.009)	0.224 (0.010)	0.020 (–0.006 to 0.046)
Initial surgery costs	3,300 (0)	3,300 (0)	–
Follow-up surgery costs	895 (188)	358 (99)	537 (121 to 954)
Primary healthcare costs (other than surgery)	5,395 (999)	4,476 (811)	920 (–1,603 to 3,441)
Secondary healthcare costs	3,369 (854)	3,099 (881)	270 (–2,135 to 2,676)
Medication costs	4 (1)	5 (1)	–1 (–2 to 1)
Unpaid productivity costs	64 (15)	57 (13)	7 (–32 to 46)
Informal care costs	1,799 (532)	1,688 (407)	109 (–1,204 to 1,422)
Total costs			
healthcare perspective	13,195 (1,381)	11,490 (1,327)	1,706 (–2,166 to 5,458)
societal perspective	15,056 (1,488)	13,235 (1,387)	1,822 (–2,109 to 5,753)

There were no significant differences in utility scores and QALYs between the 2 surgical approaches in the crude analyses. Costs in all categories were similar in both treatment groups (Table 2), with the exception of the follow-up surgery costs, which were significantly higher in the PLA (mainly due to the higher number of dislocations; see Tol et al. [10]). There were no significant differences in total healthcare and societal costs between groups, but a tendency for higher costs in the PLA group.

### Cost-effectiveness

**Figure 2:**
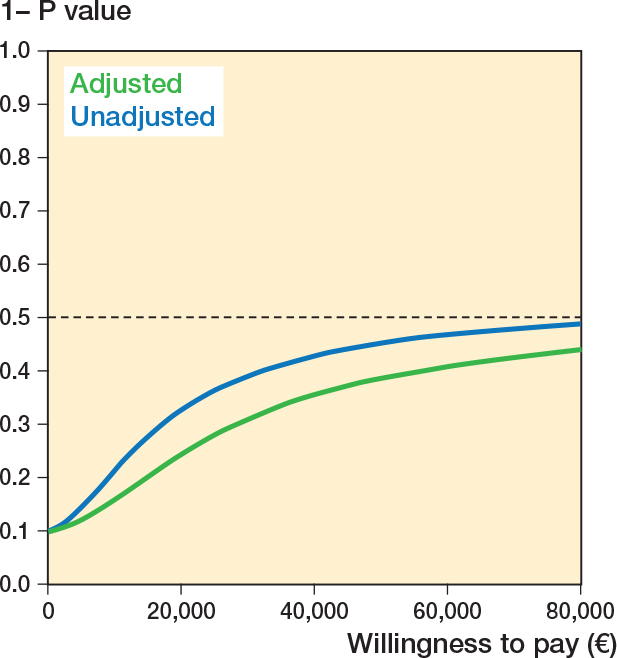
Cost-effectiveness acceptability curves (CEAC) for both analyses, societal perspective. The curves indicate the probability of PLA being cost-effective compared with DLA, conditional on the willingness-to-pay threshold.

**Figure 3:**
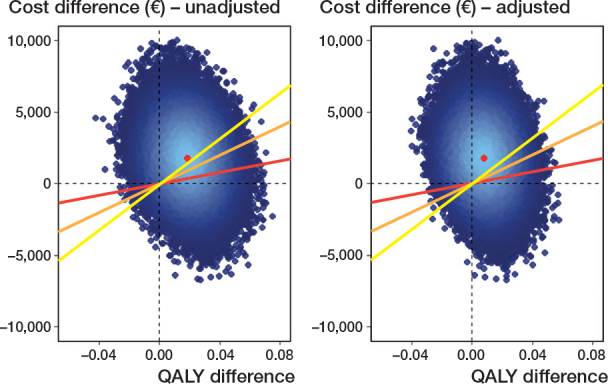
Cost-effectiveness plane for unadjusted and adjusted analysis, societal perspective. The yellow, orange, and red lines represent, respectively, the willingness-to-pay thresholds of €20,000, €50,000, and €80,000 per QALY. For each line, the proportion of points below the line is the probability of PLA being cost-effective at the corresponding threshold.

From both the healthcare and societal perspective, the ICERs showed that PLA was—on average—“more costly” and “more effective” than DLA. At willingness-to-pay thresholds of €20,000, €50,000, and €80,000 per QALY, the point estimates for the NMBs were negative, although none were statistically significant (Table 3). Figure 3 shows that, at a willingness-to-pay of €0 per QALY, PLA had a 0.10 probability of being cost-effective compared with DLA. This means that if decision-makers are not willing to pay anything per QALY gained, the probability of PLA being cost-effective compared with DLA is only 10%. This probability increased with higher willingness-to-pay thresholds but remained below 0.50 across the full range with a joint uncertainty concerning the cost and QALY difference between PLA and DLA (Figure 2). The cost-effectiveness results, as well as the calculation of the probability of cost-effectiveness, are further illustrated in Figure 3.

**Table 3:** Results of regression analyses (adjusted for baseline measurements)

Perspective	ΔCosts (CI)	ΔQALY (CI)	NMB (€20,000) (CI)	NMB (€50,000) (CI)	NMB (€80,000) (CI)	ICER
Healthcare	1,508 (–1,744 to 4,760)	0.009 (–0.014 to 0.032)	–1,331 (–5,590 to 2,929)	–1,064 (–10,589 to 8,460)	–798 (–15,791 to 14,195)	169,970
Societal	1,583 (–1,972 to 5,137)	0.009 (–0.014 to 0.032)	–1,405 (–5,756 to 2,945)	–1,139 (–10,710 to 8,432)	–873 (–15,900 to 14,153)	178,444

CI = 95% confidence interval, ICER = incremental cost-effectiveness ratio, NMB = net monetary benefit.

## Discussion

We aimed to estimate the relative cost-effectiveness of PLA and DLA in the treatment with cemented hemiarthroplasty in adults suffering an acute femoral neck fracture. The results suggest no conclusive evidence of any differences between the surgical approaches with respect to costs, QALYs, and cost-effectiveness.

We found no difference in HRQoL between the approaches, as previously found in the primary analyses of the RCT [10]. There were significantly higher follow-up surgery costs in the PLA group, which can be attributed to a much higher rate of dislocations, which was 5.5% and 0.4%, respectively [10]. In line with this finding, we found a tendency for higher secondary healthcare costs (e.g., specialists, hospital expenses, such as an emergency visit) after PLA compared with DLA; however, this difference was not significant. In the Netherlands, the reduction of a dislocated hip is frequently performed as a closed reduction in the emergency room with the use of procedural sedation. The costs of this procedure are not adequately documented in Dutch hospitals. For an emergency room consultation, a standard fee is charged, independent of which treatments and anesthesia and team were needed. Therefore, in this study, the secondary healthcare costs for PLA may have been underestimated.

A direct comparison of our results with the literature is challenging due to the lack of research on the comparative costs and cost-effectiveness of PLA and DLA in the context of hemiarthroplasty. It is noteworthy that the average healthcare costs and utility scores we found for hip fracture patients in the Netherlands were somewhat lower than those estimated in 2 recent studies [2,4]. These differences may have resulted from variations in methodology, patient population, and the element of chance due to the limited sample sizes in both the aforementioned studies and ours.

### Strengths

To date, this is the first economic evaluation of a randomized controlled trial comparing the 2 most used surgical approaches for hemiarthroplasty. Another strength of the study is that we included patients suffering from dementia. Dementia is often an exclusion criteria in clinical trials, even though patients with dementia present a substantial part of the population of patients with a hip fracture [25]. We increased the generalizability of the results by including them.

### Limitations

One limitation of this study is the substantial proportion of missing data. The observations from self-assessments through questionnaires were partially missing for many patients, as is commonly the case in trial-based cost-effectiveness studies [24]. There was no missing data regarding dislocations, reoperations, and admission to the ER, which was used for the follow-up surgery and secondary healthcare costs. Although multiple imputation can mitigate the bias caused by informative missingness to some extent, we still encountered very large standard errors in the estimates, making it difficult to draw strong conclusions from the results of our study. A further limitation concerns the generalizability of our findings, as not all eligible patients were randomized. We lack detailed information on the specific reasons for non-participation, which limits our ability to confirm that the data represents an unbiased sample of the target patient population. Nevertheless, a comparison with the Dutch Arthroplasty Register indicates that the baseline characteristics in our sample are comparable to those of the broader patient population. Another limitation is the relatively short follow-up duration of only 6 months, which restricts the ability to capture the longer-term effects of the intervention on healthcare utilization and costs [12]. As some complications may occur later, future studies should evaluate cost-effectiveness over longer follow-up, ideally combining trial-based data with model-based extrapolations. The sample size calculation was based on a minimal detectable change value (0.08) derived from the EQ-5D-3L, as evidence for the EQ-5D-5L was not yet available at the study’s initiation. The EQ-5D-5L generally shows improved measurement properties, and more recent evidence suggests a slightly higher MID for improved health states (0.11) [26]. However, this does not affect our interpretation, as the observed difference in HRQoL (0.009) was well below both thresholds.

### Conclusion

We found no evidence of a difference in cost-effectiveness between PLA and DLA for hemiarthroplasty following acute femoral neck fractures in adult patients. We therefore suggest that, from an economic viewpoint, the 2 surgical approaches should be treated as interchangeable.

### Supplementary material

An Appendix showing how QALYs, CEAC, and ICER were calculated is available on the article homepage, doi: 10.2340/17453674.2025.45056

## Supplementary Material



## Notes

Research design: JE, MT, RP, NW, TG, HW, MH, JvD. Data collection: MT, AR, TG, MS, MH, RW. Data analysis: JE, JvD. Project management: JE, MT. Drafting/revising article: JE, MT, RP, NW, TG, MS, HW, JG, MH, JvD.

Handling co-editors: Cecilia Rogmark

*Acta* thanks Fredrk Borgström and Torbjørn Berge Kristensen for help with peer review of this manuscript.
