# Supplementary material for: Comparing the posterolateral and the direct lateral approach for cemented hemiarthroplasty after femoral neck fracture: a cost-effectiveness analysis
Source: Acta Orthop. 2025 Dec 18;96:914–9. doi: 10.2340/17453674.2025.45056 (PMC12715378; doi:10.2340/17453674.2025.45056)
Supplement: Supplementary file 1 [file ActaO-96-45056-s1.pdf]

Supplementary data

Calculation of QALYs

As is customary in cost-effectiveness analyses [27], we calculate the total QALY as a weighted average of the measured QoL scores. Let $QALY_i$ be the QALY of individual i, $U_{i,k}$ the measured utility at the follow-up point k, with $U_{i,0}$ the baseline utility. Let D_k be the time that has passed at follow-up point k since the beginning of the study (expressed in years), with $D_0 = 0$. Then

$$QALY_i = \sum_{k=1}^{K} \frac{1}{2}\left(U_{i,k-1} + U_{i,k}\right)\left(D_k - D_{k-1}\right)$$

In our study, patients' quality of life scores were measured at baseline, after 3 months, and finally after 6 months. So the above formula reduces to

$$QALY_i = \frac{1}{2}\left[\left(U_{i,0} + U_{i,1}\right)\left(\frac{3}{12} - 0\right) + \left(U_{i,1} + U_{i,2}\right)\left(\frac{6}{12} - \frac{3}{12}\right)\right]$$

$$= \frac{1}{8}\left(U_{i,0} + 2U_{i,1} + U_{i,2}\right)$$

Calculation and interpretation of the cost-effectiveness acceptability curve (CEAC)

Let Δ_c and Δ_q be the expected differences in costs and QALYs between the 2 treatment options. Let λ be the maximum price that society is willing to pay per additional QALY gained in exchange for that price. Then the intervention is deemed to be cost-effective if and only if $\lambda\Delta_q - \Delta_c > 0$. The left side of this inequality is known as the net monetary

benefit (NMB) and can be computed as a function of λ and the mean differences in costs and QALYs [28].

Let $\widehat{\Delta_c}$ and $\widehat{\Delta_q}$ be the point estimates for the treatment effects; let $\widehat{\mathbf{V}} = \begin{bmatrix} \widehat{Var}(\widehat{\Delta_c}) & \widehat{Cov}(\widehat{\Delta_c}, \widehat{\Delta_q}) \\ \widehat{Cov}(\widehat{\Delta_c}, \widehat{\Delta_q}) & \widehat{Var}(\widehat{\Delta_q}) \end{bmatrix}$ be the estimated covariance matrix. Then the point estimate and variance estimate of the NMB are given as

$$\widehat{NMB}(\lambda) = \lambda\widehat{\Delta_q} - \widehat{\Delta_c}$$

$$\widehat{Var}\left(\widehat{NMB}(\lambda)\right) = \lambda^2\widehat{Var}\left(\widehat{\Delta_q}\right) + \widehat{Var}\left(\widehat{\Delta_c}\right) - 2\lambda\widehat{Cov}(\widehat{\Delta_c}, \widehat{\Delta_q})$$

Based on the point estimates and the estimated covariance matrix of the treatment effects, we compute the quantity

$$A(\lambda) = \phi\left(\frac{\widehat{NMB}(\lambda)}{\left[\widehat{Var}\left(\widehat{NMB}(\lambda)\right)\right]^{1/2}}\right)$$

where $\phi(.)$ is the cumulative distribution function of the standard normal random variable. In the classical statistical paradigm, $A(\lambda)$ is 1 minus the P value for the one-sided hypothesis test $H_0: \lambda\Delta_q - \Delta_c \leq 0$ versus $H_1: \lambda\Delta_q - \Delta_c > 0$ [23]. In contemporary practise, $A(\lambda)$ is often instead interpreted as the "probability of cost-effectiveness," so $P\left(\lambda\Delta_q - \Delta_c > 0\right)$. Even though it is often left implicit, such an interpretation is only possible under a Bayesian paradigm [29]. We do not prescribe either framework; readers may interpret the estimates as they wish.

Regardless of interpretation, it is customary in economic evaluations not to commit to a single willingness-to-pay λ, but instead to compute $A(\lambda)$ for a range of

possible values of λ and plot the 2 against each other. This plot is known as the cost-effectiveness acceptability curve (CEAC).

Incremental cost-effectiveness ratio (ICER)

The estimate for the ICER is calculated through the formula

$$ICER = \frac{\widehat{\Delta_c}}{\widehat{\Delta_q}}$$

While the ICER is widely used in cost-effectiveness research, it suffers from numerous severe problems. Its interpretation is difficult, and it is impossible to construct meaningful confidence intervals that quantify the uncertainty around the point estimate of the ICER. See the detailed discussion in Willan and Briggs [23], chapter 4. In consequence, we focus on the NMB approach outlined in the previous section.
